# Enhanced In Vitro Antiviral Activity of Ivermectin-Loaded Nanostructured Lipid Carriers against Porcine Epidemic Diarrhea Virus via Improved Intracellular Delivery

**DOI:** 10.3390/pharmaceutics16050601

**Published:** 2024-04-29

**Authors:** Xiaolin Xu, Shasha Gao, Qindan Zuo, Jiahao Gong, Xinhao Song, Yongshi Liu, Jing Xiao, Xiaofeng Zhai, Haifeng Sun, Mingzhi Zhang, Xiuge Gao, Dawei Guo

**Affiliations:** 1 Engineering Center of Innovative Veterinary Drugs, Center for Veterinary Drug Research and Evaluation, MOE Joint International Research Laboratory of Animal Health and Food Safety, College of Veterinary Medicine, Nanjing Agricultural University, 1 Weigang, Nanjing 210095, China; 2 Academy for Advanced Interdisciplinary Studies, Nanjing Agricultural University, Nanjing 210095, China; 3 Jiangsu Key Laboratory of Pesticide Science, College of Sciences, Nanjing Agricultural University, 1 Weigang, Nanjing 210095, China

**Keywords:** ivermectin, antivirus, nanostructured lipid carriers, porcine epidemic diarrhea virus, intracellular delivery

## Abstract

Porcine epidemic diarrhea virus (PEDV) is an acute enteric coronavirus, inducing watery diarrhea and high mortality in piglets, leading to huge economic losses in global pig industry. Ivermectin (IVM), an FDA-approved antiparasitic agent, is characterized by high efficacy and wide applicability. However, the poor bioavailability limits its application. Since the virus is parasitized inside the host cells, increasing the intracellular drug uptake can improve antiviral efficacy. Hence, we aimed to develop nanostructured lipid carriers (NLCs) to enhance the antiviral efficacy of IVM. The findings first revealed the capacity of IVM to inhibit the infectivity of PEDV by reducing viral replication with a certain direct inactivation effect. The as-prepared IVM-NLCs possessed hydrodynamic diameter of 153.5 nm with a *zeta* potential of −31.5 mV and high encapsulation efficiency (95.72%) and drug loading (11.17%). IVM interacted with lipids and was enveloped in lipid carriers with an amorphous state. Furthermore, its encapsulation in NLCs could enhance drug internalization. Meanwhile, IVM-NLCs inhibited PEDV proliferation by up to three orders of magnitude in terms of viral RNA copies, impeding the accumulation of reactive oxygen species and mitigating the mitochondrial dysfunction caused by PEDV infection. Moreover, IVM-NLCs markedly decreased the apoptosis rate of PEDV-induced Vero cells. Hence, IVM-NLCs showed superior inhibitory effect against PEDV compared to free IVM. Together, these results implied that NLCs is an efficient delivery system for IVM to improve its antiviral efficacy against PEDV via enhanced intracellular uptake.

## 1. Introduction

Swine diarrhea is one of the main infectious diseases affecting the pig industry, among which viral diarrhea has the highest incidence and causes the most serious damage [1]. Coronaviruses are important pathogens causing diarrhea in piglets and mainly include porcine epidemic diarrhea virus (PEDV), porcine infectious gastroenteritis virus (TGEV), porcine delta coronavirus (PDCoV), and porcine acute diarrhea syndrome coronavirus (SADS-CoV). Currently, PEDV and TGEV are the most widely distributed viral pathogens of porcine diarrhea [2]. PEDV is a member of the genus α-coronavirus, a single-stranded, positive-stranded RNA virus with a capsid, which can cause acute diarrhea, vomiting, and dehydration in piglets, with a lethality rate of up to 90%, inducing significant economic losses to the pig industry worldwide [3,4,5]. Available PEDV vaccines include inactivated and attenuated vaccines, but these diseases are still frequent in the pig breeding industry, partly due to the emergence of new mutant strains [6]. With the risk of emerging or re-emerging diseases, there is an urgent need to develop new antiviral compounds [4,7,8].

Recently, drug repurposing has become an attractive option for treating emerging diseases, as developing new effective drugs against a particular pathogen is time-consuming and expensive [9]. In contrast, approved substances are easily available, and their potential side effects are well characterized [10]. Drug repurposing is a strategy for converting an FDA-approved or investigational drug from its original use to a new use [11]. The greatest benefit of repurposed drugs is the omission of critical and time-consuming drug development phases, which significantly reduces the time required to produce effective antiviral drugs [12].

Ivermectin (IVM) is an FDA-approved, broad-spectrum, high-efficiency, low-toxicity antiparasitic drug [13,14]. It is a kind of hexadecameric ring macrolide drug, and its biological activity is broad [15]. Studies have shown that it has potential therapeutic effects against viruses [16]. In vitro investigations have demonstrated that IVM could effectively restrict infection caused by a variety of RNA and DNA viruses, including HIV-1, dengue virus (DENV), related flaviviruses, influenza A, and Venezuelan equine encephalitis virus (VEEV) [17,18,19,20,21]. Recent studies have indicated that it is a potent inhibitor of SARS-CoV-2 [22]. However, the low solubility of IVM in water, only 6-9 micrograms per milliliter, and the poor bioavailability limit its clinical applications [23,24]. Nanostructured lipid carriers are a new generation of lipid nanoparticles consisting of a mixture of solid and liquid lipids [25]. It is a cutting-edge nano-delivery system that enhances the solubility, stability, and bioavailability of the encapsulated bioactive compounds by protecting them from adverse environmental conditions and regulates their release by enabling them to exert their active effects at the right time and site [26,27,28].

In this study, we determined that IVM could inhibit the infectivity of PEDV, and then, formulated nanostructured lipid carriers could be used strategically to tackle the low solubility and the poor bioavailability of IVM. The results showed that NLCs could enhance the antiviral activity of IVM by improving intracellular delivery. This study aimed to verify the potential of IVM-NLC as an alternative promising therapeutic drug for PEDV.

## 2. Materials and Methods

### 2.1. Chemical, Cells, and Viruses

Ivermectin (IVM, 91%) was purchased from the China Institute of Veterinary Drug Control (Beijing, China). African green monkey epithelial cells (Vero) and PEDV strain CV777 (GenBank Accession No. KT323979) were maintained in the laboratory.

### 2.2. Preparation of IVM-NLCs

The high-shear-ultrasound and high-pressure homogenization methods were used to prepare IVM-NLCs [29,30]. Briefly, a certain amount of oleic acid (OA) (Aladdin, Shanghai, China), palmitic acid (PA, Aladdin, Shanghai, China), Tween 20 (Aladdin, Shanghai, China), and IVM was mixed as the oil phase and heated up to 70 °C until complete melting. In parallel, poloxamer 188 (Yunhong Chemical Preparations and Accessories Technology Corporation, Shanghai, China) was dissolved in water as the aqueous phase and heated at the same temperature. Then, the aqueous phase was poured into the oil phase under magnetic stirring at 700 rpm. Subsequently, the mixture was homogenized at 11,000 rpm for 5 min with a High Shear Dispersion Emulsifying Machine (FM200, IKA, Staufen, Germany), and then treated by probe sonication at 300 W for 20 min with an Ultrasonic Cell Disruptor (JY96-II, Scientz, Ningbo, China). The obtained oil-in-water (O/W) emulsion was rapidly transferred into cold water under high-shear conditions for 1 min and cooled to form the NLCs. The hot O/W system was cycled four times in a high-pressure homogenizer (AH-BASIC, ATS, Suzhou, China) at 700 bar to obtain IVM-NLCs in bulk.

### 2.3. Characterization of IVM-NLCs

#### 2.3.1. Hydrodynamic Diameter (HD), Polydispersity Index (PDI), and Zeta Potential (ZP)

Prior to measurement, all the samples were diluted appropriately with deionized water. The HD, PDI, and ZP of IVM-NLCs were characterized using dynamic light scattering (DLS) with the Zetasizer (Malvern Instruments Ltd., Malvern, Worcestershire, UK).

#### 2.3.2. Transmission Electron Microscopy (TEM)

The morphology of the IVM-NLCs was observed by TEM (Tecnai 12, Philips, Amsterdam, The Netherlands). Briefly, the diluted IVM-NLCs were dropped onto 300-mesh copper grids. After drying, the samples were negatively stained for 2 min using a 2% (*w*/*v*) phosphotungstic acid solution. The dried samples were then subjected to TEM analysis.

#### 2.3.3. X-ray Diffraction (XRD) and Fourier Transform Infrared Spectroscopy (FT-IR)

PA, IVM, physical mixtures of PA and IVM, lyophilized NLCs, and IVM-NLC powder were studied using XRD (D8 Advance, Bruker AXS, Karlsruhe, Germany) to analyze the changes in the crystal structure of IVM during NLC formation. In addition, the above samples were placed in a crucible and compacted onto slides. Subsequently, XRD with Cu/Kα radiation source were scanned at 4°/min in the range of 5°~50° under the conditions of 40 kV/40 mA. After that, PA, IVM, physical mixture of IVM and PA, lyophilized NLCs, and IVM-NLC powder IRs were recorded using an FT-IR spectrometer (IS5&N380, Nicolet, Waltham, MA, USA). Prior to FT-IR spectroscopy, the samples were mixed with potassium bromide (KBr) in a ratio of 1:150 (*w*/*w*) and pressed into thin slices using a high-pressure hydraulic press. The prepared flake samples were then placed on an FT-IR spectrometer and scanned under a wave number range from 4000 cm^−1^ to 500 cm^−1^.

#### 2.3.4. Entrapment Efficiency (EE) and Drug Loading (DL)

The EE and DL of IVM-NLCs were determined by ultrafiltration centrifugation combined with high-performance liquid chromatography (HPLC). The diluted IVM-NLCs were added to methanol, vortexed, and sonicated to break the emulsion. The supernatant was centrifuged, and the weight of IVM (W_total_) in the emulsion was determined by HPLC. In addition, the dilution of IVM-NLCs was placed in ultra-filtration centrifuge tube (MWCO: 100 kDa, Millipore, Bilrika, MA, USA) at high speed, and the weight of free IVM (W_free_) in the emulsion was determined by HPLC. The DL and EE of IVM-NLCs were calculated using the following formulas:

EE (%) = (W_total_ − W_free_)/W_total_

DL (%) = (W_total_ − W_free_)/W_NLCs_

where W_total_ is the total content of applied IVM in NLCs, W_free_ is the amount of free IVM in the supernatant phase, and W_NLCs_ is the content of the lipid used in the preparation of the IVM-NLCs.

### 2.4. Cell Culture

Vero cells were grown in Dulbecco’s Modified Eagle’s Medium (DMEM, Gibco, Waltham, MA, USA) with 1% penicillin–streptomycin (HyClone, Logan, UT, USA) and 5% Fetal Bovine Serum (FBS) (Gibco, CA, USA). Cells were placed in a humidified cell culture incubator with 5% CO_2_ at 37 °C.

### 2.5. Cell Viability Evaluation

The cytotoxicity of IVM and IVM-NLCs was tested in Vero cells using the Cell Counting kit-8 (CCK-8, KeyGen Biotech Co., Ltd., Nanjing, China) method. Vero cells were seeded in a 96-well plate overnight at a density of 10,000 cells per well in 100 μL fresh medium. After plating, cells were treated with IVM and IVM-NLCs for 24 h and 48 h. Subsequently, CCK-8 (10 μL/well) was added to each well and incubated at 37 °C for 1 h, and the OD values at 450 nm were measured. Cell viability was calculated using the following formula: cell viability (%) = (OD_treated_ − OD_blank_)/(OD_untreated_ − OD_blank_) × 100%.

### 2.6. In Vitro Cellular Uptake

Qualitative and quantitative assessment of in vitro cellular uptake of coumarin-6 (C6) in Vero cells using fluorescence microscopy (Thermo Fisher, Waltham, MA, USA) and flow cytometry (BD Biosciences, New York, NY, USA), respectively. Briefly, Vero cells were seeded into a 6-well culture plate at the density of 2 × 10^5^ cells per well and incubated overnight. The cells were then treated with free C6 or C6-NLCs, and cells without any treatment were used as a control. After 4 h, cells were washed with cold PBS, fixed in 4% paraformaldehyde for 10–15 min, labeled with DAPI, and visualized by fluorescence microscopy. In addition, to further investigate the cellular uptake process of C6 and C6-NLCs, after the termination of uptake, treated cells were separated with 0.25% trypsin, centrifuged at 1000 rpm for 5 min, and suspended in PBS, and fluorescent signals in cells were analyzed by flow cytometry.

### 2.7. TCID_50_ Assay

To determine the 50% tissue culture infectious dose (TCID_50_), PEDV samples were diluted 10-fold with maintenance solution and used to inoculate Vero cells. Briefly, Vero cells were seeded in a 96-well plate overnight. Then, the medium was discarded, washed twice with maintenance solution, and 100 μL diluted virus solution per well and maintenance solution was added to the negative control wells. The cell culture plates were placed in an incubator at 37 °C and incubated for 1 h. After that, the old maintenance solution was discarded, and 100 μL of maintenance solution was added to the incubator for continued cultivation and monitoring of CPE. TCID_50_/mL was calculated according to the method of Reed–Muench.

### 2.8. Antiviral Assay

Cells were infected with PEDV at multiplicity of infection (MOI) of 0.05. One hour later, the inoculum was removed, and the cells were washed with serum-free DMEM. Subsequently, cells were incubated separately with DMEM, NLCs, IVM, and IVM-NLCs. The antiviral effect of NLCs, IVM, and IVM-NLCs on PEDV infection was then evaluated by quantitative real-time polymerase chain reaction (RT-qPCR), Western blot, and indirect immunofluorescence assay (IFA).

There exists a possible target for inhibition at each stage of viral infection [31,32]. To determine at which stage IVM blocked infection, IVM as well as PEDV were added to cells with different treatments:

Inactivation assay: The mixture of IVM with PEDV was placed at 37 °C for 3 h. Vero cells were infected with the pretreated PEDV for 1 h. After incubation, the supernatant was discarded and replaced with a maintenance solution without any drugs to continue the culture. Total RNA was extracted and quantified by RT-qPCR.

Attachment assay: Vero cells were pre-cooled at 4 °C, followed by incubation with 5 μM IVM for 1 h, and then infection with PEDV for 1 h at 4 °C. After discarding the supernatant, the cells were washed twice with pre-cooled serum-free DMEM. Total RNA was extracted and quantified by RT-qPCR.

Adsorption assay: After pre-cooling at 4 °C, Vero cells were infected with PEDV at 4 °C for 1 h. After discarding the supernatant and washing with pre-cooled serum-free DMEM, the cells were treated with 5 μM IVM in DMEM containing 2% FBS for 1 h at 37 °C. Total RNA was extracted and quantified by RT-qPCR.

Replication assay: Vero cells were infected with PEDV for 1 h at 37 °C. To remove non-adsorbed virus particles, the cells were washed with pre-cooled serum-free DMEM, incubated with DMEM for 4 h, and then treated with 5 μM IVM for 12 h. Total RNA was extracted and quantified by RT-qPCR.

Release assay: Vero cells were infected with PEDV for 10 h at 37 °C. The supernatant was discarded, and after washing with serum-free DMEM, the cells were incubated separately with IVM in DMEM containing 2% FBS. Total RNA was extracted and quantified by RT-qPCR.

### 2.9. One-Step Growth Curve

Vero cells at 80–90% confluence were infected with 0.05 MOI of PEDV. Subsequently, the control DMEM, NLCs, IVM, and IVM-NLCs were added and subjected to further incubation at 37 °C. The TCID_50_ was recorded at 1, 4, 8, 12, 24, 36, 48, 60, and 72 hpi according to the Reed–Muench method. PEDV titers were calculated, and viral growth curves were plotted by determining TCID_50_ at different time points.

### 2.10. RT-qPCR

*RT-qPCR* was based on SYBR Green method. The PEDV total RNA was extracted from the cells in a 6-well plate using RNAiso Plus (Takara, Tokyo, Japan) according to the manufacturer’s protocol. The concentration and purity of total RNA were assessed by Nanodrop (Thermo Fisher Scientific, Waltham, MA, USA). The total RNA was reverse transcribed into cDNA using cDNA synthesis kit (Vazyme, Nanjing, China) and stored at −20 °C. The target genes were evaluated in triplicate using SYBR qPCR Master Mix (Vazyme, Nanjing, China). Absolute fluorescence was quantitatively referenced as described in [33].

### 2.11. Western Blot Analysis

Vero cells were cultured to approximately 80–90% confluence in 6-well plates, and infected with 0.05 MOI of PEDV. After 1 h of infection, the cell monolayers were incubated with control DMEM, NLCs, IVM, and IVM-NLCs (5 μM) for 24 h, and then treated with lysis buffer (100 μL/well) to extract total protein, followed by the quantification of protein concentration using bicinchoninic acid (BCA) protein assay kit according to the manufacturer’s instructions (Solarbio, Beijing, China). Sodium dodecyl sulfate (SDS) loading buffer was added to the collected cell extracts and boiled for 10 min. Equivalent amounts of proteins were loaded and electrophoresed on 12% sodium dodecyl sulfate polyacrylamide gel electrophoresis (SDS-PAGE), and then transferred to nitrocellulose filter (NC) membranes followed by blocking with 5% skim milk for 2 h. Subsequently, the expression of the PEDV N protein was determined. The expression of GAPDH was investigated to represent the same amounts of protein sample loading.

### 2.12. IFA

The inhibitory effect of NLCs, IVM, and IVM-NLCs against PEDV in Vero cells was further evaluated by immunofluorescence. Vero cells in 24-well plates were infected with PEDV at 0.05 MOI. After 1 h of incubation, free viruses were removed by extensive rinsing. Then, the cells were incubated with control DMEM (containing 2% FBS) or NLCs, IVM, and IVM-NLCs, respectively. Twenty-four hours later, cells were fixed with 4% formaldehyde for 15 min. After permeabilization with 0.1% Triton X-100, the cells were incubated with the mouse monoclonal antibody against the PEDV N protein (1:200 dilution) at 4 °C overnight and washed three times with phosphate-buffered solution with Tween 20 (PBST) for 10 min each. Then, the cells were incubated with FITC-conjugated goat anti-mouse antibody (1:200 dilution) for 1 h, and counterstained with DAPI at room temperature for 10 min. After washing three times, the photographs were obtained by fluorescence microscopy.

### 2.13. Determination of Reactive Oxygen Species (ROS) Generation

ROS level was assessed using a 2′,7′-dichlorofluorescein diacetate (DCFH-DA)-based ROS assay kit (Nanjing Kaiji Biotechnology Co., Ltd., Nanjing, China). Briefly, well-grown Vero cells were inoculated in 12-well plates, and an inoculum of 0.05 MOI of PEDV (500 μL/well) was added to Vero cells grown to 80–90% fusion. After 1 h of infection, the cell monolayer was rinsed and then covered with NLCs, IVM, and IVM-NLCs, respectively. After that, the cells were incubated with DCFH-DA for 20 min at 37 °C in the dark. The fluorescence changes in cells in each group were observed under a fluorescence microscope (Ex = 488 nm, Em = 507 nm) (Thermo Fisher, MA, USA).

### 2.14. Mitochondrial Membrane Potential (MMP) Analysis

JC-1 assay kit (KeyGen Biotech Co., Ltd., Nanjing, China) was used to detect MMP changes. Well-grown Vero cells were inoculated in 12-well plates, and an inoculum of 0.05 MOI of PEDV (500 μL/well) was added to Vero cells grown to 80–90% fusion. After 1 h of infection, the cell monolayer was rinsed and then covered with NLCs, IVM, and IVM-NLCs. Subsequently, the cells were washed with PBS. JC-1 (10 μg/mL) was added to each sample and incubated at 37 °C in the dark for 15 min. The fluorescence changes in cells in each group were observed under a fluorescence microscope (green fluorescence, Ex = 488 nm, Em = 507 nm; red fluorescence, Ex = 525 nm, Em = 590 nm) (Thermo Fisher, MA, USA).

### 2.15. Apoptosis Assay

Apoptosis rate of PEDV-infected Vero cells was assessed with Annexin V-FITC/PI double staining kit (KeyGen Biotech Co., Ltd., Nanjing, China) according to the manufacturer’s instructions. The cells were seeded into 6-well plates and then stained with Annexin V-FITC and PI in dark. Cells were analyzed by flow cytometry (Ex = 488 nm, Em = 525 nm). Cell-Quest software (Becton Dickinson, San Jose, CA, USA) was employed to analyze the data.

### 2.16. Statistical Analysis

All data are presented as mean ± standard deviation (SD), and experiments were conducted in triplicate. GraphPad Prism 8.0 software was used for statistical analysis. Differences between the two groups were assessed using *t*-test (mean ± SD). When comparing several groups, one-way analysis of variance (ANOVA) was employed. Statistics were applied to differences with * *p* < 0.05 and ** *p* < 0.01.

## 3. Results and Discussion

### 3.1. IVM Inhibited the Infectivity of PEDV

**Figure 1:**
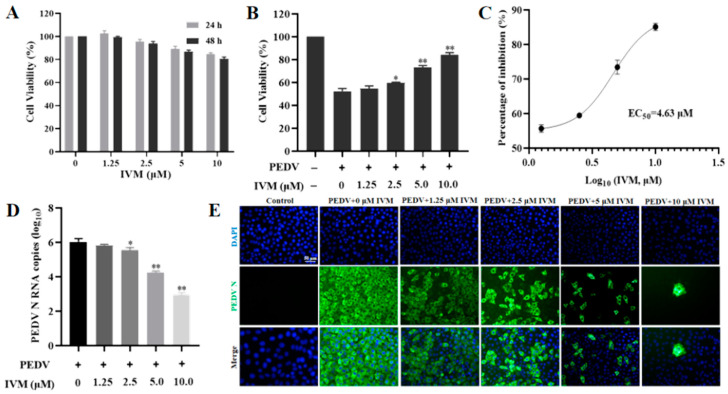
IVM inhibited the infectivity of PEDV. (**A**) Cytotoxicity of Vero cells treated with different concentrations of IVM at the appointed time via CCK-8 assay. Medium containing 0.05% DMSO (*v*/*v*) served as control. (**B**) Antiviral activity of IVM was measured by CCK-8 assay. (**C**) EC_50_ of IVM was calculated with GraphPad Prism 8.0. (**D**) PEDV N RNA copies of Vero cells infected with PEDV after different treatments of IVM by RT-qPCR. (**E**) Indirect immunofluorescence assay of PEDV-infected Vero cells after treatment with different concentrations of IVM or without treatment. Blue, DAPI; green, FITC-conjugated goat anti-mouse antibody. Scale bar = 50 μm. Error bars represent the standard deviation from three repeated experiments. The mean value was calculated by the one-way analysis of variance (ANOVA) (mean ± SD, n = 3). * *p* < 0.05, ** *p* < 0.01, compared with the PEDV group.

Numerous studies have recently reported the antiviral properties of IVM, which could impede viruses by hindering nuclear input that was reliant on specific IMPα/β1-dependent viral proteins [2]. This study investigated the antiviral activity of IVM against PEDV in vitro. Prior to assessing its inhibitory potency, cytotoxicity assays were conducted on Vero cells. The results indicated that cell viability remained above 80% at concentrations ranging from 0 to 10 µM (Figure 1A). After excluding cytotoxicity, the inhibitory effect of IVM on PEDV-infected Vero cells was evaluated. Specifically, Vero cells were infected with 0.05 MOI of PEDV and subsequently treated with varying concentrations of IVM. Furthermore, a discernible difference was observed among the virus control group after 2.5 or 5 μM IVM treatment, and cell viability could reach more than 80% after 10 μM IVM treatment, as illustrated in Figure 1B. We calculated the concentration for 50% of maximal effect (EC_50_) value, which reflected the concentration of IVM required to abolish infectious virus particle production by 50%. The EC_50_ value for IVM following infection with PEDV in Vero cells was 4.63 μM (Figure 1C). Furthermore, the administration of IVM at concentrations of 1.25, 2.5, 5, and 10 μM resulted in a reduction of PEDV RNA N copies at 12 h post-infection from 6.1 to 5.8, 5.5, 4.8, and 3.8 lg copies, respectively, in comparison to the untreated cohort (Figure 1D). Notably, the PEDV nucleocapsid (N) protein, an RNA-binding protein crucial for the virus life cycle, could serve as a precise and early diagnostic target for PEDV infection [34,35]. The inhibitory effect of IVM on PEDV proliferation was verified by detecting the expression level of PEDV N protein. Specifically, the infected cells were incubated with different concentrations of IVM. As shown in Figure 1E, at 24 hpi, a strong fluorescent signal was observed within the PEDV-infected Vero cells treated with DMSO by indirect immunofluorescence. However, we observed a significant and concentration-dependent difference between untreated infected cells and IVM-treated infected cells, which was in accordance with the growth curves of the virus.

### 3.2. Effect of IVM in Diverse Stages of PEDV Life Cycle

**Figure 2:**
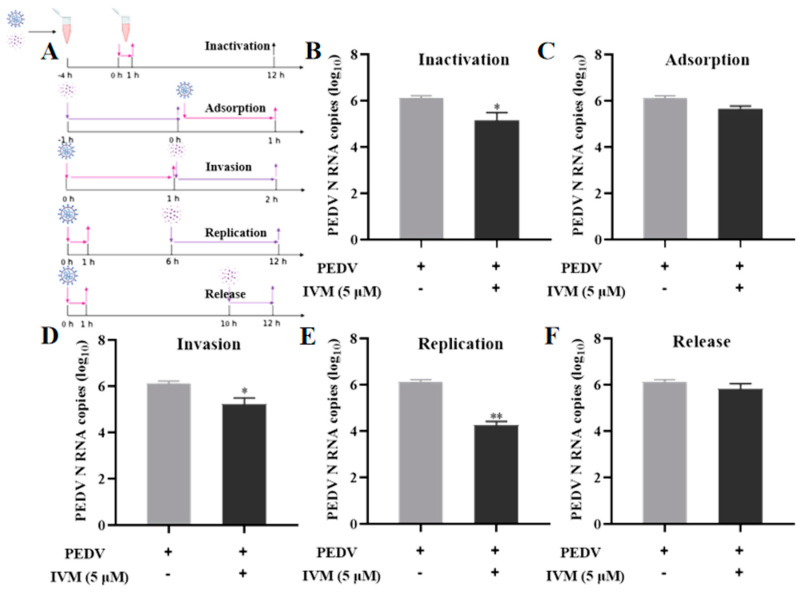
IVM treatment at multiple stages of inhibition of PEDV proliferation. (**A**) Schematic diagram of the effect of IVM on the replication cycle of PEDV. (**B**) Effect of IVM on direct inactivation of PEDV. Effect of IVM on the (**C**) adsorption, (**D**) invasion, (**E**) replication, and (**F**) release processes of infected cells. The mean value was calculated by the *t*-test (mean ± SD, n = 3). * *p* < 0.05, ** *p* < 0.01, compared with the PEDV group.

The present study investigated the underlying mechanism of the antiviral properties of IVM by analyzing their impact on the proliferation of PEDV during its replication cycle. The schematic diagram is shown in Figure 2A. The direct inactivation potential of IVM on PEDV was initially evaluated, and the results demonstrated a 10-fold reduction in the number of PEDV via RT-qPCR depicted in Figure 2B. In the adsorption process of PEDV, there was no significant difference between the experimental group and the control group in terms of their suppression effect on PEDV adsorption (Figure 2C). In the invasion process of PEDV, the results revealed that IVM treatment decreased the infectious virus titer by about 10-fold relative to the control group (Figure 2D), implying IVM had slight influence on PEDV invasion. In addition, as shown in Figure 2E, IVM reduced the number of PEDV N RNA copies by nearly 10^2^-fold, implying that IVM may suppress PEDV mainly via inhibiting PEDV replication. In addition, the effect of IVM on the release of PEDV progeny is shown in Figure 2F. There was no noticeable difference in the virus titers of PEDV compared to the control group, suggesting that IVM had no inhibitory effect on the release of PEDV progeny. To sum up, IVM exerted its suppressive effect on PEDV primarily by inhibiting virus invasion and replication with a certain direct inactivation effect in vitro.

### 3.3. Characterization of IVM-Loaded Nanostructured Lipid Carriers

**Figure 3:**
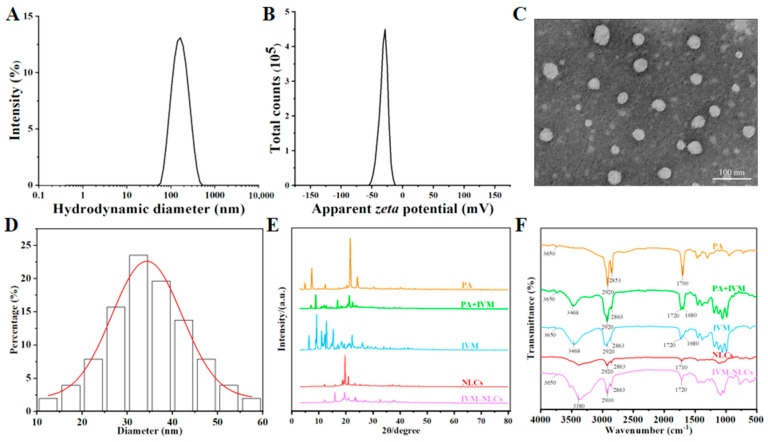
Characterization of the optimized IVM-NLCs. (**A**) Hydrodynamic diameter (HD) and (**B**) *zeta* potential (ZP) of the IVM-NLCs were determined by DLS. (**C**) The morphology of the IVM-NLCs was observed by TEM, and (**D**) the size distribution was obtained via analysis of the particles from several TEM images. Scale bar = 100 nm. (**E**) X-ray diffraction (XRD) spectra and (**F**) Fourier transform infrared (FT-IR) spectra for IVM-NLCs, NLCs, physical mixture, IVM, and PA were shown.

The prepared IVM-NLCs through the high-pressure homogenization technique exhibited characteristics of a homogeneous, opaque, and milky white liquid with high fluidity. The hydrodynamic diameter (HD) and *zeta* potential (ZP) of the IVM-NLCs, as illustrated in Figure 3A,B, demonstrated a narrow normal distribution. The as-prepared IVM-NLCs possessed an HD of 153.5 ± 0.80 nm with a polydispersity index (PDI) of 0.153 ± 0.007, indicating a high degree of particle size distribution homogeneity (Table S1). ZP was also a critical factor to evaluate the stability of colloidal dispersion. In general, stable dispersion of a nanoparticle system was achieved when the absolute value of ZP exceeded 30 mV due to electrical repulsion [36]. The ZP of IVM-NLCs was −31.5 ± 0.569 mV, indicating favorable stability. The morphology of IVM-NLCs was examined using transmission electron microscopy (TEM), revealing spherical or ellipsoidal particles with uniform size distribution and no observed agglomeration (Figure 3C). The mean distribution size of IVM-NLCs was determined to be 39.54 ± 9.17 nm (Figure 3D). Notably, the particle size of IVM-NLCs was significantly smaller than the HD. The reason for this disparity is that dynamic light scattering (DLS) provides an indirect measurement of particle size by detecting the fluctuation in scattered light intensity due to Brownian motion in a hydrated state, whereas TEM requires the sample to be in a dry state during testing [37,38]. DL and EE were important parameters for evaluating the preparation of NLCs. Increasing EE could enhance drug efficacy and reduce adverse drug reactions. Increasing DL could lead to a more stable formulation, while reducing the use of excipients and thus their potential toxicity. The EE and DL of the prepared IVM-NLCs measured by HPLC were 95.72 *±* 0.30% and 11.17 *±* 0.75%, respectively (Table S1). The prepared IVM-NLCs was based on the laboratory preparation of IVM-SLNs [30]. Compared with IVM-SLNs, IVM-NLCs had smaller HD and more uniform distribution, which improved EE and DL.

The conversion of drugs from a crystalline to an amorphous state had been found to enhance drug loading and improve the stability of nanodrug delivery systems [39]. The X-ray diffraction pattern was used for crystallographic analysis [40]. As shown in Figure 3E, apparent diffraction peaks could be observed near 10°, 15°, and 20° in the pattern of IVM, indicating that IVM was a crystalline structure. The diffraction peaks observed in the XRD patterns of IVM were still observed in the physical mixture of PA and IVM. The sharp diffraction of IVM-NLCs disappears significantly near 10°, while the characteristic diffraction peaks persist near 15° and 20° but unlike IVM, which suggest that the IVM has reacted in the NLCs, weakening its intermolecular forces, and the crystallinity of the IVM has also weakened. In addition, the diffraction peaks observed in NLCs were consistent with IVM-NLCs, indicating that IVM was dispersed in NLCs in an amorphous form. The FT-IR spectra of PA, IVM, physical mixture of IVM and PA, freeze-dried IVM-NLC powder, and NLC powder are displayed in Figure 3F. The characteristic absorption peak of C=C at 1680 cm^−1^ in IVM-NLCs disappeared and the characteristic peak of C-O-C stretching vibration of IVM group at 1050–1200 cm^−1^ was significantly reduced; the sharp peak at 3650 cm^−1^ for IVM is a stretching vibration of the alcohol hydroxyl group, which also occurs in IVM-PA mixtures and IVM-NLCs; IVM had the O-H stretching vibration peak of hydroxyl group at 3468 cm^−1^, and so did the physical mixture of IVM and PA; while the O-H stretching vibration peak of IVM-NLCs was blue-shifted, suggesting that the binding between drug and carrier occurred, marking the successful preparation of nanostructured lipid carriers; the waveforms of IVM and IVM-NLCs were basically similar, indicating that the lipid carriers did not change the skeletal structure of IVM, and IVM was wrapped in the lipid carriers in non-crystalline form. The XRD and FT-IR results were in agreement, and IVM was transformed from crystals to amorphous in IVM-NLCs, encapsulated into the nanostructured lipid matrix in an amorphous state.

### 3.4. NLCs Improved Cellular Uptake of IVM

**Figure 4:**
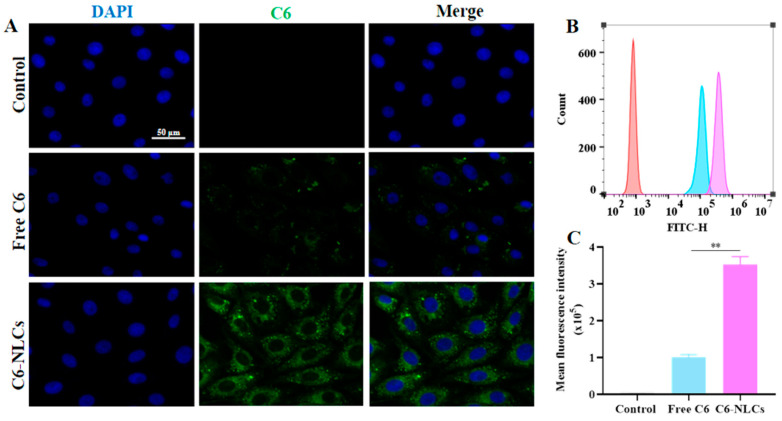
Effect of NLCs on the cellular uptake of coumarin-6 (C6) in Vero cells. (**A**) Representative fluorescence microscope images of various preparations uptake in Vero cells. (**B**) Cell uptake was determined by flow cytometry of C6 in Vero cells after treatment with free C6 and C6-NLCs and (**C**) mean intracellular fluorescence intensity. Scale bar = 50 μm. The mean value was calculated by the *t*-test (mean ± SD, n = 3). ** *p* < 0.01, for C6-NLCs vs. free C6.

Since the virus is parasitized within the host cells, increasing intracellular drug uptake can improve antiviral efficacy [41,42]. In order to evaluate the in vitro biocompatibility of IVM-NLCs and IVM, cytotoxicity assays were conducted by incubating Vero cells with varying concentrations of IVM or IVM-NLCs for 24 and 48 h, respectively. The results for Vero cells are presented in Figure S1. The relative survival of cells after exposure to IVM-NLCs (0–10 μM) for 24 and 48 h was greater than 80%, indicating that IVM-NLCs have good biocompatibility. The experimental findings demonstrated a significant enhancement in the biocompatibility of IVM-NLCs in comparison to IVM. Additionally, the cytotoxicity of IVM-NLCs on Vero cells exhibited a dose- and time-dependent relationship. The fluorescence microscopy results depicted in Figure 4A indicated that C6-NLCs displayed more pronounced fluorescence signals than free C6. Moreover, the fluorescence intensity exhibited by C6-NLCs was significantly higher than that of free C6, as depicted in Figure 4B. Furthermore, the mean fluorescence intensity of C6-NLCs in Vero cells was 3.7-fold greater than that of free C6, as determined through flow cytometry analysis in Figure 4C. These findings collectively suggested that NLCs hold potential as a delivery carrier for augmenting the cellular uptake of IVM.

### 3.5. NLCs Enhanced the Antiviral Activity of IVM against PEDV

**Figure 5:**
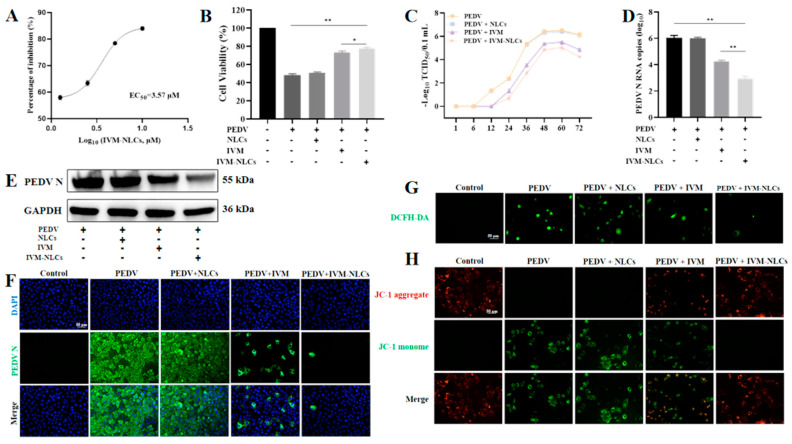
Anti-PEDV activity of IVM-NLCs on Vero cells. (**A**) EC_50_ of IVM-NLCs was calculated with GraphPad Prism 8.0. (**B**) Antiviral activity of NLCs, IVM, and IVM-NLCs (5.0 μM) measured by CCK-8 assay. (**C**) One-step growth curve of virus after treatment or without treatment with NLCs, IVM, and IVM-NLCs. (**D**) PEDV N RNA copies of Vero cells infected with PEDV after the treatment with NLCs, IVM, and IVM-NLCs by RT-qPCR. (**E**) Western blot analysis of the expression level of PEDV N protein under the treatment of NLCs, IVM, and IVM-NLCs. (**F**) Indirect immunofluorescence assay of infected cells. Blue, DAPI; green, FITC-conjugated goat anti-mouse antibody. (**G**) Cellular reactive oxygen species (ROS) level in infected cells post different treatments. (**H**) Mitochondrial membrane potential (MMP) in infected cells post different treatments. Scale bar = 50 µm. Error bars represent the standard deviation from three repeated experiments. The mean value was calculated by the one-way analysis of variance (ANOVA) (mean ± SD, n = 3). * *p* < 0.05, ** *p* < 0.01.

The present study initially assessed the cell viability of Vero cells infected with PEDV using IVM-NLCs through the CCK-8 assay (Figure S1). The inhibitory effect of IVM-NLCs on PEDV-infected Vero cells is shown in Figure S2. The EC_50_ value for IVM-NLCs following infection with PEDV in Vero cells was 3.57 μM (Figure 5A). The EC_50_ value for IVM-NLCs was lower than IVM and confirmed that IVM-NLCs exhibited higher antiviral activity than IVM. The results illustrated in Figure 5B indicated that NLCs improved the viability of Vero cells at 48 h post-treatment in comparison to free IVM. To assess the impact of NLCs, IVM, and IVM-NLCs on PEDV replication, the one-step growth curve was generated through measuring the PEDV titer following treatment with 5 μM of each agent. In Figure 5C, at 12 hpi, PEDV began to proliferate, with a period of rapid proliferation from 24 hpi to 48 hpi and a peak viral titer of 10^6.5^ TCID_50_/0.1 mL at 60 hpi. After 60 h, PEDV proliferation decreased due to cell collapse. Compared to the negative control group, significant viral titer inhibition was observed in cells treated with IVM-NLCs. Therefore, the changes in the titer of PEDV verified that IVM-NLCs indeed possessed superior antiviral activity against viral replication.

Additionally, RT-qPCR analysis revealed that IVM-NLCs exhibited a greater reduction in PEDV N RNA copies, as expected in Figure 5D. To validate the inhibitory impact of IVM-NLCs on the proliferation of PEDV, we assessed the expression level of PEDV N protein. Specifically, the Western blot assay demonstrated a significant reduction in the expression level of PEDV N protein upon treatment with IVM-NLCs (Figure 5E). Although the downregulation of PEDV N protein expression was observed in both IVM and IVM-NLCs treatment groups, a more pronounced effect was observed in the IVM-NLC-treated group. Additionally, we incubated infected cells with NLCs, IVM, and IVM-NLCs. At 24 hpi, a strong fluorescent signal was observed in PEDV-infected Vero cells by IFA. However, a marked difference was observed in the number of infected cells in the IVM-NLCs treated group versus the untreated PEDV-infected group, as indicated by green fluorescence (Figure 5F). These findings collectively indicated that NLCs may enhance the antiviral activity of IVM against PEDV.

Reactive oxygen species (ROS) were toxic byproducts of cellular metabolism, primarily generated by mitochondria in mammalian cells, and were involved in regulating multiple physiological functions of cells [43]. We investigated the effect of IVM-NLCs on ROS production during PEDV infection. Our findings, as depicted in Figure 5G, revealed a substantial increase in DCF fluorescence intensity in infected Vero cells. Conversely, cells treated with IVM-NLCs exhibited a significant reduction in ROS generation compared to those treated with IVM alone. The results indicated that ROS was involved in the antiviral effect of IVM-NLCs. ROS caused mitochondrial membrane damage, resulting in MMP disorder [44]. In normal cells, JC-1 emitted red fluorescence. In contrast, in PEDV-infected Vero cells, JC-1 exhibited green fluorescence, which indicated that PEDV had disrupted the mitochondrial membrane potential of Vero cells, resulting in its decline. Following treatment with IVM-NLCs, the mitochondrial membrane potential was notably restored (Figure 5H). In summary, compared with IVM, IVM-NLCs could improve the inhibition of MMP damage and impede intracellular ROS accumulation in infected Vero cells.

### 3.6. Effect of IVM-NLCs on the Apoptosis Rate in PEDV-Infected Vero Cells

**Figure 6:**
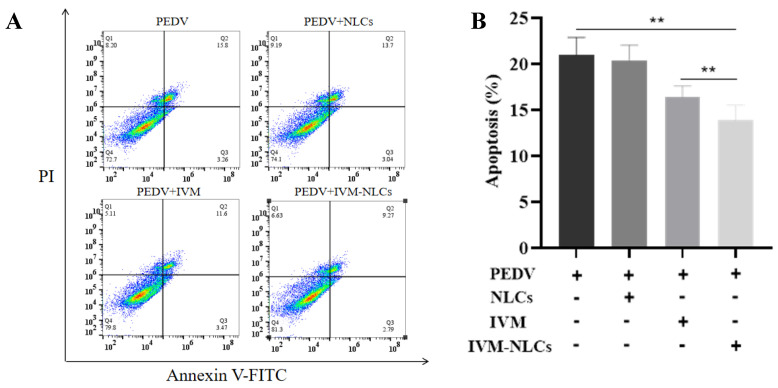
Apoptosis of PEDV-infected Vero cells after treatment with IVM-NLCs. (**A**) Apoptosis assay was performed in PEDV-infected Vero cells treated with NLCs, IVM (5 μM), and IVM-NLCs (5 μM). (**B**) The graph represents the percentage of apoptosis in Vero cells after treatment. The mean value was calculated by the one-way analysis of variance (ANOVA) (mean ± SD, n = 3). ** *p* < 0.01.

To investigate the mechanism of PEDV inhibition by ivermectin, an AnnexinV-FITC/PI kit was used to detect the apoptosis of the cells by flow cytometry. The results showed that PEDV could induce apoptosis rate of 20.9 ± 1.89% (Figure 6A), and the apoptosis rates of IVM- and IVM-NLC-treated PEDV-infected groups were significantly reduced to 16.4 ± 1.17 and 13.9 ± 1.59 (Figure 6B), which indicated that they play an important biological function in PEDV-induced apoptosis. These findings suggested that IVM-NLCs reduced ROS accumulation in PEDV-infected Vero cells by improving the inhibition of MMP damage, thereby reducing apoptosis in infected cells.

## 4. Conclusions

Herein, the inhibitory effect of IVM on PEDV in vitro was first demonstrated. IVM could inhibit PEDV by the direct inactivation of viral particles and the inhibition of the replication phase. Subsequently, IVM-NLCs were successfully developed with excellent physicochemical properties and improved solubility, it could serve as a promising nanocarrier for IVM with an increased solubility and enhanced pharmacological efficacy. According to biological tests, IVM-NLCs exhibited stronger antiviral activity against PEDV than free IVM and reduced PEDV-induced mitochondrial dysfunction, which prevented ROS generation and improved viability of infected Vero cell. Moreover, IVM-NLCs also reduced PEDV-induced cell apoptosis rate. In view of the in vitro results, it would be necessary to carry out in vivo tests as soon as possible, to explore its potential in the clinical treatment of PEDV. Consequently, IVM-NLCs were demonstrated to be a potential drug against PEDV, which might provide a basis for the development of novel drugs to antagonize PEDV.

## Data Availability

The data presented in this study are available in this article and Supplementary Material.

## Supplementary Materials

The following supporting information can be downloaded at: https://www.mdpi.com/article/10.3390/pharmaceutics16050601/s1, Figure S1: Cytotoxicity of Vero cells treated with different concentration of IVM at the appointed time via CCK-8 assay; Figure S2: Antiviral activity of IVM-NLCs was measured by CCK-8 assay; Table S1 Characterization of as-prepared IVM-NLCs.
