# Supplementary material for: Enhanced In Vitro Antiviral Activity of Ivermectin-Loaded Nanostructured Lipid Carriers against Porcine Epidemic Diarrhea Virus via Improved Intracellular Delivery
Source: Pharmaceutics. 2024 Apr 29;16(5):601. doi: 10.3390/pharmaceutics16050601 (PMC11125651; doi:10.3390/pharmaceutics16050601)
Supplement: Supplementary file 1 [file pharmaceutics-16-00601-s001.zip › pharmaceutics-2961884-supplementary.pdf]

Supporting Information

Xiaolin Xu [1], Shasha Gao [1], Qindan Zuo [1], Jiahao Gong [1], Xinhao Song [1], Yongshi Liu [1], Jing Xiao [1], Xiaofeng Zhai [1,2], Haifeng Sun [1], Mingzhi Zhang [3], Xiuge Gao [1] and Dawei Guo [1,*]

[1] Engineering Center of Innovative Veterinary Drugs, Center for Veterinary Drug Research and Evaluation, MOE Joint International Research Laboratory of Animal Health and Food Safety, College of Veterinary Medicine, Nanjing Agricultural University, 1 Weigang, Nanjing 210095, China
[2] Academy for Advanced Interdisciplinary Studies, Nanjing Agricultural University, Nanjing 210095, China
[3] Jiangsu Key Laboratory of Pesticide Science, College of Sciences, Nanjing Agricultural University, 1 Weigang, Nanjing 210095, China
* Correspondence: gdawei0123@njau.edu.cn; Tel.: +86-25-8439-6215; Fax: +86-25-8439-8669

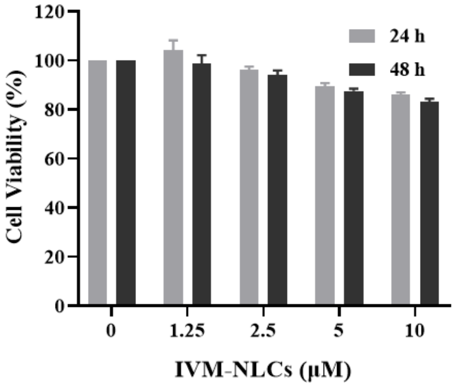

Figure S1 Cytotoxicity of Vero cells treated with different concentration of IVM at the appointed time via CCK-8 assay. Error bars represent the standard deviation from three repeated experiments.

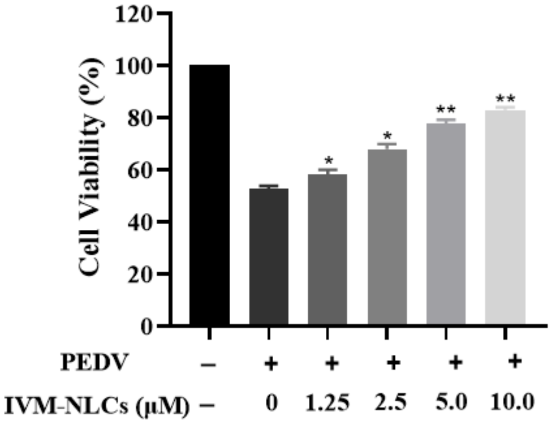

Figure S2 Antiviral activity of IVM-NLCs was measured by CCK-8 assay. Error bars represent the standard deviation from three repeated experiments. The mean value was calculated by the one-way analysis of variance (ANOVA) (mean ± SD, n = 3). *p < 0.05, **p < 0.01 , ns, non-significant difference, compared with the PEDV group.

Table S1 Characterization of as-prepared IVM-NLCs

Indicators	HD (nm)	PDI	ZP (mV)	EE (%)	DL (%)
IVM-NLCs	153.5 ± 0.80	0.153 ± 0.007	−31.5 ± 0.569	95.72 ± 0.30%	11.17 ± 0.75%
